# Two Babies, Two Bonds: Challenges in Attachment Relationships in Twins

**DOI:** 10.7759/cureus.76422

**Published:** 2024-12-26

**Authors:** Daniela Simões, Tiago Soares, Graça Fernandes

**Affiliations:** 1 Department of Child and Adolescent Psychiatry, Local Health Unit of Western Lisbon, Lisbon, PRT; 2 Department of Child and Adolescent Psychiatry, Local Health Unit of Santo António, Porto, PRT

**Keywords:** attachment style, feeding difficulties, identical twins, insecure ambivalent/resistant attachment, prematurity, selective fetal growth restriction, strange situation paradigm

## Abstract

The attachment relationship constitutes the first emotionally significant affective bond, usually between the infant and the mother, serving as a model for subsequent relationships. It is considered a vital component of social and emotional development in the early years and an important early indicator of infant mental health. In twins, the attachment process may exhibit unique characteristics, influenced by the dual parenting dynamic and the individual needs of each baby. With this article, we intend to explore the challenges of infant-caregiver relationships and the attachment process in twins through a brief, non-systematic literature review regarding a clinical case of a 21-month-old female born from a twin pregnancy complicated by selective fetal growth restriction (sFGR), prematurity, and very low birth weight. The patient was initially suspected of displaying an insecure ambivalent/resistant attachment style, differing from her twin sister. Studies have shown a high concordance in attachment style between twin pairs, although a higher prevalence of insecure ambivalent/resistant attachment style compared to the general population. This may reflect challenges commonly presented in twin pregnancies and the perinatal period, such as sFGR, prematurity, and feeding difficulties, which increase parental stress that may impair early attachment between parents and infants. Attachment is not a fixed bond. Early identification and intervention in infant-caregiver relationship difficulties within this vulnerable group can help mitigate the challenges of dual parenting, fostering secure bonds and promoting healthy infant development.

## Introduction

Primary caregiving relationships and the surrounding relational environment form the foundation for the development and functioning of infants and young children. The attachment relationship constitutes the first emotionally significant affective bond, usually between the infant and the mother, serving as a model for subsequent relationships [1].

Attachment is most often assessed in the early years of life with a procedure known as the Strange Situation Procedure, developed by Mary Ainsworth [1]. Conducted in a controlled setting, this procedure involves a series of short episodes where the young child experiences separations and reunions with the caregiver and interactions with a stranger. Based on the child's responses - such as distress during separation, comfort-seeking during reunion, and willingness to explore the environment - it is possible to classify the attachment style between the child and caregiver: secure attachment (distress when separated but comforted upon return, demonstrating confidence to explore the environment), insecure-avoidant attachment (minimal distress and avoidance upon reunion, often suppressing distress and engaging superficially with the environment), insecure ambivalent/resistant attachment (intense distress with mixed reactions upon reunion, exhibiting hesitation to explore due to preoccupation with the caregiver’s availability), and disorganized attachment (contradictory, confused behaviors, with inconsistent willingness to explore) [1]. The absence of a secure attachment compromises the normal psychoaffective development of the infant and child, acting as a vulnerability factor for psychopathology [1].

The attachment process in twins may exhibit unique characteristics, influenced by the dynamics of dual parenting and the individual needs of each baby. Some theorists have proposed that a mother can only attach to one infant at a time, suggesting that discrepancies in attachment styles between twin siblings might be common [2]. However, both clinical practice and recent research indicate that parents of twins strive to treat their babies equally, often fearing preferential treatment and feeling guilty if they cannot meet both babies' needs in the same way [2]. Understanding these specificities is essential for developing interventions that promote the well-being of both the infants and their parents.

This article aims to explore the challenges in infant-caregiver relationships in the case of twins and their impact on attachment styles through a brief, non-systematic literature review prompted by a clinical case. Articles for this review were selected from the PubMed database, including only recent studies published in English that were directly relevant to the topic and the clinical case.

## Case presentation

The patient is a 21-month-old female (corrected age: 19 months) living with her parents and twin sister. She was referred to the Early Childhood Psychiatry Clinic for feeding difficulties, sleep disturbances, and behavioral changes. She attended the first consultation accompanied by her father.

During an open interview, the father described that the patient has been eating less than expected for her age since birth, shows little interest in food, and frequently throws tantrums at mealtimes, which has led to great exhaustion and family tension over time. The parents expressed concern about the patient’s weight since birth, as she was born with a very low birth weight (1120 grams). However, she is now in the 15th percentile for both weight and height, and her growth has remained appropriate for her percentile. No feeding difficulties were reported at daycare, where, unlike at home, she does not resist during meals. Regarding sleep, she has slept alone in her own room since six months old, but only falls asleep in the presence of one of her parents. She wakes up during the night for a bottle of milk, with no additional awakenings until morning. This routine is maintained by her parents, as any change causes her to cry, waking her sister. The patient sleeps about 10 hours per night and takes a nap after lunch at daycare. Regarding her demeanor, oppositional tendencies towards her parents and instances of hetero-aggression directed at her sister during moments of frustration have been noted over the past few months.

Her medical history highlights a monochorionic/diamniotic twin pregnancy. In the third trimester, selective fetal growth restriction (sFGR) was detected in the first fetus (the clinical case patient), necessitating the mother’s hospitalization from 24 weeks of gestation. This period was marked by significant parental distress. Delivery was spontaneous at 32 weeks, with no need for resuscitation. The newborn was admitted to the neonatal intensive care unit (NICU) for 29 days due to prematurity, sFGR with very low birth weight, and neonatal jaundice. Her twin sister was hospitalized for the same period due to prematurity, low birth weight, and neonatal jaundice. Parents expressed fatigue and insecurity while caring for the twins, particularly regarding feeding. Breastfeeding was not possible due to maternal hypogalactia. The patient and her twin sister were fed with infant formula until five months, when complementary feeding began, with no signs of food selectivity. Psychomotor development has been described as adequate to date. She is followed up in the Pediatric Sleep Outpatient Clinic for the aforementioned sleep disturbances and receives melatonin treatment at a daily dose of 1 mg. She also attends the Neurodevelopment Outpatient Clinic due to prematurity. No relevant family medical or psychiatric history was reported.

On examination, the patient appeared clean, well-groomed, and cared for, with no dysmorphic features. She entered the consultation room in her father’s arms, displaying a reserved attitude but with modulated eye contact. She initiated exploration of the available toys after some time, exhibiting mainly sensorimotor and functional play and frequently referencing her father. Her receptive language appeared preserved, as she followed simple commands given by her father and vocalized isolated words throughout the consultation. No stereotypies were observed. The Strange Situation Paradigm (a procedure adapted from Mary Ainsworth’s Strange Situation applied during the first consultation of toddlers aged 12-26 months in our Early Childhood Psychiatry Clinic) revealed a behavioral pattern consistent with an insecure ambivalent/resistant attachment style (Table 1). It is worth noting that the patient was tired and easily irritable, having started crying immediately before the application of this technique.

**Table 1:** Comparison of the patient’s two observational results under the Strange Situation Paradigm

Strange Situation Paradigm	Patient’s first observation (attachment figure: father)	Patient’s second observation (attachment figure: mother)
Exploration or play: toddler plays without active involvement of the caregiver; duration: three minutes.	The patient sought contact with the father, not engaging in exploration or play.	The patient explored the room and played, seeking regular contact with the mother.
Separation episode: caregiver leaves the room, and an observer (the stranger) interacts with the toddler; duration: three minutes.	The patient showed intense distress due to the father's absence, requiring premature interruption.	The patient protested the mother's absence but was briefly comforted by the stranger.
Reunion episode: caregiver returns to the room.	The patient ran towards the father, expressing irritation and seeking comfort.	The patient received the mother with pleasure and relief, seeking her comfort.
Free play: toddler engages in play with the caregiver; duration: three minutes.	The patient remained irritable and unavailable for play, constantly seeking father's comfort.	The patient became comforted and engaged in play with the mother.

Her twin sister also began follow-up in the Early Childhood Psychiatry Clinic on the same day, due to feeding difficulties very similar to those of the clinical case patient. The twin sister was accompanied to the consultation by their mother. The application of the Strange Situation Paradigm in this case revealed a behavioral pattern consistent with a secure attachment style.

Given the differing results between the sisters, the mother was asked to attend the second consultation with the clinical case patient so that the technique could be repeated and a comparison of the twins’ attachment styles with the same attachment figure could be made. In the second consultation, which occurred two weeks after the first one, the patient demonstrated a behavioral pattern consistent with a secure attachment style (Table 1).

Subsequently, the patient underwent an occupational therapy evaluation, which revealed low body awareness and avoidance of proprioceptive activities. She was referred for sensory integration therapy with weekly sessions and continued monthly early childhood psychiatry consultations focused on the parent-infant relationship. Gradually, the patient started eating larger quantities of food, experiencing fewer tantrums and less family tension during mealtimes. Aggression toward her parents and sister also decreased. Currently, she no longer wakes up every night and continues to take melatonin at a dose of 1 mg per day.

## Discussion

Emerging research on the attachment process in twins has demonstrated increased challenges in infant-caregiver relationships. These include reduced sensitivity and involvement of mothers in interactions with twin infants, frequent reports of sadness and guilt due to insufficient time with each one individually, and heightened stress levels caused by the additional demands of caregiving and the higher likelihood of medical complications and sleep/feeding disturbances in these infants [3-6]. A recent study investigated differences in mothers' bonding with each baby in a twin pair, finding that most mothers (76%) reported feeling equally attached to both babies. Discrepancies in bonding with each baby were associated with higher levels of maternal depression and parental stress, and lower relationship satisfaction [2].

These findings align with earlier research showing a high concordance in attachment style among twin siblings and a moderate to high concordance in attachment style across non-twin siblings [7,8]. However, there appears to be a higher prevalence of insecure ambivalent/resistant attachment styles in twins compared to the general population [9]. Using the presented clinical case, we highlight three challenges frequently observed during twin pregnancies and the perinatal period that may contribute to these findings: sFGR, prematurity, and feeding difficulties.

sFGR

sFGR is defined as the weight of one fetus being below the 10th percentile for gestational age and occurs in at least 10% of monochorionic twin pregnancies. sFGR is associated with higher rates of perinatal morbidity and mortality and long-term motor, cognitive, and psychosocial developmental alterations [10,11].

A study examined the attachment process in monochorionic twins with sFGR, finding a higher prevalence of insecure ambivalent/resistant attachment style in monochorionic twins with sFGR (36%) compared to the general population (16%). They also identified a trend toward concordance in attachment style within the same twin pair [9]. The attachment process begins during pregnancy. Parents of monochorionic twins with sFGR often experience a difficult pregnancy, facing increased risks of perinatal loss and, in many cases, the possibility of selective reduction of the smaller fetus. Heightened levels of uncertainty, sadness, anxiety, and stress-commonly identified in these parents can impair early bonding with their infants. Concordance in attachment style within the same twin pair suggests a greater influence of the pregnancy course rather than the individual characteristics of the infants [9].

Prematurity

Twin pregnancies are a significant risk factor for prematurity (birth before 37 weeks of gestation), which can negatively impact primary caregiving relationships and, in turn, attachment styles [12-14].

The global prevalence of preterm live birth is approximately 15%. In Portugal, about 8% of all live births are preterm, and about 1.2% of infants are born very preterm (VPT; born between 28 and 32 weeks) or extremely preterm (EPT; born before 28 weeks) [14]. Despite significant advances in medicine over recent decades, prematurity remains a public health challenge and one of the leading causes of mortality in children under five [14]. To promote survival and reduce morbidity, VPT or EPT (VEPT) infants often undergo life-saving but intrusive and painful interventions in the NICU, which limit parental contact and consequently the establishment of infant-caregiver relationships [14]. These early challenges seem to contribute to increased distress and dysregulation in infants, potentially affecting how they learn to regulate attention and emotions, interact with attachment figures, and adjust to life outside the NICU [14].

A longitudinal study conducted in Portugal with 213 infants and their mothers showed that secure attachment style was more prevalent in term infants, while insecure ambivalent/resistant attachment style was more common in VEPT infants [13]. The quality of mother-infant interaction at three and nine months (corrected age) was related to attachment style at 12 months and varied by gestational age. VEPT infants were found to be more difficult and less passive, while their mothers were less sensitive and responsive [13]. Differences in maternal sensitivity and interaction quality may represent adaptations to the infants' characteristics rather than ineffective parenting. However, initially, adaptive maternal behaviors may become maladaptive if they fail to adjust to the evolving needs and capacities of the infant [12].

Another Portuguese study aimed to identify specific relational, familial/demographic, and perinatal predictors of attachment style in a sample of 63 VEPT infants, in which approximately one-third of mothers had social/family risk factors [14]. No association was found between any single risk factor and attachment style at 12 months. However, when two or more social/family risk factors were combined, a significant association emerged, indicating that both relational and social contextual factors contribute to attachment style in this biologically vulnerable sample [14]. These findings align with other research showing that psychosocial risk status appears to play a larger role than prematurity in infant-mother interaction quality [15].

Feeding difficulties

Preterm infants are at increased risk of feeding difficulties, which may persist throughout childhood. Owing to their lower oromotor tone, poor sucking-swallowing-breathing coordination, and disturbed sleep-wake cycles, they have prolonged nasogastric tube feeding and delayed oral feeding independence [16]. All these variables compromise the successful initiation and continuation of breastfeeding, resulting in its early cessation. Parents frequently feel pressured to adhere to rigid feeding schedules and closely monitor growth, exposing them to high-stress levels [16]. Caregivers' emotional state and the immature behaviors of preterm infants, including less perceptible hunger cues, often lead to conflictual feeding interactions [16].

Compared to term infants, preterm infants exhibited fewer positive interactions with their mothers during feeding periods and more feeding refusal behaviors between 18 and 24 months [17]. Similarly, another study showed that mothers of preterm infants experienced higher levels of anxiety, sadness, and stress, were less responsive to their infants' hunger cues, displayed a more intrusive approach, and provided less autonomy during feeding [18]. Feeding difficulties in early childhood thus negatively impact the well-being of infants and their caregivers, posing a challenge to their relationships and, consequently, the attachment process [16].

Based on the findings of our research, several important clinical implications emerge. The data emphasize the need for early intervention practices and family policies to empower and enable parents of twins to actively engage in sensitive, rewarding, reciprocal, and affectional relationships with their infants from birth [2,13]. For maximum effectiveness, early intervention should start as early as pregnancy, preparing parents for their future caregiving roles and offering them social/economic support [13].

## Conclusions

Although the initial suspicion that the patient presented a different attachment style from her twin sister was not confirmed, this case study allowed us to delve into the unique and challenging characteristics of primary caregiving relationships and the attachment process in twins.

The data presented underscore the importance of providing parental counseling by healthcare professionals to parents of twins regarding the challenges inherent in dual parenting, both during pregnancy and throughout the first year of their infants' lives. Since attachment is not a static process, the early identification of difficulties in primary caregiving relationships within this vulnerable group enables timely interventions. These interventions can help parents develop stress management strategies and strengthen their roles as caregivers, fostering sensitive, rewarding, and reciprocal interactions that promote the healthy development of infants.
